# Supplementary material for: Field Evaluation of Rice Lines Derived from Suakoko 8 X Bao Thai for Iron Tolerance in the South Saharan African Farming System
Source: Plants (Basel). 2024 Jun 11;13(12):1610. doi: 10.3390/plants13121610 (PMC11207341; doi:10.3390/plants13121610)
Supplement: Supplementary file 1 [file plants-13-01610-s001.zip › plants-2988716-supplementary.pdf]

Entry	Genotypes	Location	GY (kg/ha)	Location	GY (kg/ha)	Loss (%)
82	IR 88638-177-1-1-1-1-1-1	Edozighi	1351	Ibadan	977	-38
73	IR 88638-168-1-1-1-1-1-1	Edozighi	1209	Ibadan	1202	-1
89	IR 88638-183-1-1-1-1-1-1	Edozighi	1843	Ibadan	1864	1
234	IR 88638-325-1-1-1-1-1-1	Edozighi	956	Ibadan	1015	6
6	IR 88638-102-1-1-1-1-1-1	Edozighi	640	Ibadan	685	7
250	IR 88638-34-1-1-1-1-1-1	Edozighi	1608	Ibadan	1732	7
325	IR 88638-98-1-1-1-1-1-1	Edozighi	1904	Ibadan	2718	30
149	IR 88638-240-1-1-1-1-1-1	Edozighi	1187	Ibadan	1765	33
36	IR 88638-13-1-1-1-1-1-1	Edozighi	1430	Ibadan	2352	39
139	IR 88638-230-1-1-1-1-1-1	Edozighi	2855	Ibadan	4793	40
220	IR 88638-308-1-1-1-1-1-1	Edozighi	1537	Ibadan	2765	44
264	IR 88638-39-1-1-1-1-1-1	Edozighi	2803	Ibadan	5223	46
115	IR 88638-208-1-1-1-1-1-1	Edozighi	1299	Ibadan	2431	47
5	IR 88638-101-1-1-1-1-1-1	Edozighi	2161	Ibadan	4157	48
43	IR 88638-137-1-1-1-1-1-1	Edozighi	676	Ibadan	1308	48
251	IR 88638-341-1-1-1-1-1-1	Edozighi	1898	Ibadan	3690	49
255	IR 88638-345-1-1-1-1-1-1	Edozighi	2515	Ibadan	5045	50
287	IR 88638-62-1-1-1-1-1-1	Edozighi	2905	Ibadan	5973	51
146	IR 88638-236-1-1-1-1-1-1	Edozighi	1448	Ibadan	3198	55
8	IR 88638-104-1-1-1-1-1-1	Edozighi	1644	Ibadan	3856	57
267	IR 88638-41-1-1-1-1-1-1	Edozighi	2421	Ibadan	5678	57
281	IR 88638-56-1-1-1-1-1-1	Edozighi	857	Ibadan	2084	59
297	IR 88638-71-1-1-1-1-1-1	Edozighi	2301	Ibadan	5640	59
328	SUAKOKO 8	Edozighi	987	Ibadan	2467	60
136	IR 88638-227-1-1-1-1-1-1	Edozighi	1385	Ibadan	3481	60
178	IR 88638-268-1-1-1-1-1-1	Edozighi	1801	Ibadan	4527	60
39	IR 88638-133-1-1-1-1-1-1	Edozighi	2664	Ibadan	6700	60
188	IR 88638-277-1-1-1-1-1-1	Edozighi	1787	Ibadan	4495	60
163	IR 88638-254-1-1-1-1-1-1	Edozighi	2154	Ibadan	5484	61
155	IR 88638-247-1-1-1-1-1-1	Edozighi	882	Ibadan	2263	61
213	IR 88638-301-1-1-1-1-1-1	Edozighi	1240	Ibadan	3205	61
26	IR 88638-12-1-1-1-1-1-1	Edozighi	1425	Ibadan	3717	62
67	IR 88638-162-1-1-1-1-1-1	Edozighi	2140	Ibadan	5675	62
228	IR 88638-320-1-1-1-1-1-1	Edozighi	2124	Ibadan	5653	62
25	IR 88638-120-1-1-1-1-1-1	Edozighi	2203	Ibadan	5925	63
298	IR 88638-72-1-1-1-1-1-1	Edozighi	1670	Ibadan	4501	63
191	IR 88638-280-1-1-1-1-1-1	Edozighi	1078	Ibadan	2911	63
252	IR 88638-342-1-1-1-1-1-1	Edozighi	1543	Ibadan	4198	63
314	IR 88638-88-1-1-1-1-1-1	Edozighi	935	Ibadan	2558	63
95	IR 88638-190-1-1-1-1-1-1	Edozighi	2520	Ibadan	6917	64
170	IR 88638-26-1-1-1-1-1-1	Edozighi	1706	Ibadan	4694	64
304	IR 88638-78-1-1-1-1-1-1	Edozighi	1311	Ibadan	3609	64
184	IR 88638-273-1-1-1-1-1-1	Edozighi	2271	Ibadan	6443	65
310	IR 88638-83-1-1-1-1-1-1	Edozighi	1339	Ibadan	3838	65
144	IR 88638-234-1-1-1-1-1-1	Edozighi	1180	Ibadan	3445	66
50	IR 88638-145-1-1-1-1-1-1	Edozighi	1542	Ibadan	4524	66

301	IR 88638-75-1-1-1-1-1	Edozighi	2327	Ibadan	6851	66
58	IR 88638-153-1-1-1-1-1	Edozighi	2027	Ibadan	6008	66
269	IR 88638-44-1-1-1-1-1	Edozighi	2431	Ibadan	7270	67
37	IR 88638-131-1-1-1-1-1	Edozighi	1744	Ibadan	5252	67
110	IR 88638-204-1-1-1-1-1	Edozighi	2617	Ibadan	7987	67
257	IR 88638-347-1-1-1-1-1	Edozighi	1570	Ibadan	4795	67
244	IR 88638-335-1-1-1-1-1	Edozighi	1651	Ibadan	5044	67
312	IR 88638-85-1-1-1-1-1	Edozighi	2137	Ibadan	6622	68
2	BW348-1	Edozighi	1295	Ibadan	4035	68
238	IR 88638-330-1-1-1-1-1	Edozighi	1744	Ibadan	5439	68
254	IR 88638-344-1-1-1-1-1	Edozighi	2298	Ibadan	7302	69
318	IR 88638-91-1-1-1-1-1	Edozighi	2406	Ibadan	7654	69
237	IR 88638-328-1-1-1-1-1	Edozighi	1734	Ibadan	5565	69
160	IR 88638-251-1-1-1-1-1	Edozighi	1603	Ibadan	5187	69
317	IR 88638-9-1-1-1-1-1	Edozighi	1788	Ibadan	5879	70
41	IR 88638-135-1-1-1-1-1	Edozighi	1637	Ibadan	5397	70
128	IR 88638-219-1-1-1-1-1	Edozighi	2297	Ibadan	7606	70
180	IR 88638-270-1-1-1-1-1	Edozighi	1947	Ibadan	6448	70
199	IR 88638-287-1-1-1-1-1	Edozighi	2238	Ibadan	7455	70
127	IR 88638-218-1-1-1-1-1	Edozighi	2011	Ibadan	6865	71
329	WITA4	Edozighi	1277	Ibadan	4420	71
243	IR 88638-334-1-1-1-1-1	Edozighi	1877	Ibadan	6505	71
47	IR 88638-142-1-1-1-1-1	Edozighi	1850	Ibadan	6466	71
140	IR 88638-23-1-1-1-1-1	Edozighi	1811	Ibadan	6345	71
169	IR 88638-260-1-1-1-1-1	Edozighi	1589	Ibadan	5598	72
286	IR 88638-61-1-1-1-1-1	Edozighi	1589	Ibadan	5620	72
87	IR 88638-181-1-1-1-1-1	Edozighi	1930	Ibadan	6843	72
164	IR 88638-255-1-1-1-1-1	Edozighi	1897	Ibadan	6753	72
38	IR 88638-132-1-1-1-1-1	Edozighi	1563	Ibadan	5600	72
10	IR 88638-106-1-1-1-1-1	Edozighi	1327	Ibadan	4850	73
63	IR 88638-159-1-1-1-1-1	Edozighi	1694	Ibadan	6196	73
242	IR 88638-333-1-1-1-1-1	Edozighi	1963	Ibadan	7194	73
31	IR 88638-126-1-1-1-1-1	Edozighi	1383	Ibadan	5103	73
226	IR 88638-315-1-1-1-1-1	Edozighi	1697	Ibadan	6268	73
97	IR 88638-191-1-1-1-1-1	Edozighi	1541	Ibadan	5698	73
7	IR 88638-103-1-1-1-1-1	Edozighi	1325	Ibadan	4914	73
132	IR 88638-223-1-1-1-1-1	Edozighi	1040	Ibadan	3868	73
107	IR 88638-201-1-1-1-1-1	Edozighi	1871	Ibadan	6963	73
165	IR 88638-256-1-1-1-1-1	Edozighi	1478	Ibadan	5581	74
198	IR 88638-286-1-1-1-1-1	Edozighi	2056	Ibadan	7801	74
246	IR 88638-337-1-1-1-1-1	Edozighi	1496	Ibadan	5680	74
92	IR 88638-187-1-1-1-1-1	Edozighi	1517	Ibadan	5765	74
212	IR 88638-30-1-1-1-1-1	Edozighi	1693	Ibadan	6437	74
302	IR 88638-76-1-1-1-1-1	Edozighi	1636	Ibadan	6223	74
75	IR 88638-17-1-1-1-1-1	Edozighi	2077	Ibadan	7928	74
256	IR 88638-346-1-1-1-1-1	Edozighi	1568	Ibadan	5988	74
218	IR 88638-306-1-1-1-1-1	Edozighi	1500	Ibadan	5740	74

27	IR 88638-121-1-1-1-1-1-1	Edozighi	1583	Ibadan	6062	74
324	IR 88638-97-1-1-1-1-1-1	Edozighi	1961	Ibadan	7524	74
96	IR 88638-19-1-1-1-1-1-1	Edozighi	2022	Ibadan	7778	74
148	IR 88638-239-1-1-1-1-1-1	Edozighi	1403	Ibadan	5406	74
305	IR 88638-79-1-1-1-1-1-1	Edozighi	1850	Ibadan	7134	74
229	IR 88638-32-1-1-1-1-1-1	Edozighi	1491	Ibadan	5763	74
203	IR 88638-291-1-1-1-1-1-1	Edozighi	1106	Ibadan	4273	74
182	IR 88638-271-1-1-1-1-1-1	Edozighi	1518	Ibadan	5867	74
319	IR 88638-92-1-1-1-1-1-1	Edozighi	1456	Ibadan	5636	74
221	IR 88638-309-1-1-1-1-1-1	Edozighi	1571	Ibadan	6080	74
262	IR 88638-37-1-1-1-1-1-1	Edozighi	1426	Ibadan	5540	74
142	IR 88638-232-1-1-1-1-1-1	Edozighi	1662	Ibadan	6478	74
111	IR 88638-205-1-1-1-1-1-1	Edozighi	1596	Ibadan	6267	75
248	IR 88638-339-1-1-1-1-1-1	Edozighi	1855	Ibadan	7294	75
253	IR 88638-343-1-1-1-1-1-1	Edozighi	1023	Ibadan	4039	75
270	IR 88638-45-1-1-1-1-1-1	Edozighi	2276	Ibadan	8988	75
215	IR 88638-303-1-1-1-1-1-1	Edozighi	1433	Ibadan	5659	75
249	IR 88638-340-1-1-1-1-1-1	Edozighi	1488	Ibadan	5895	75
30	IR 88638-125-1-1-1-1-1-1	Edozighi	1481	Ibadan	5923	75
145	IR 88638-235-1-1-1-1-1-1	Edozighi	1348	Ibadan	5396	75
94	IR 88638-189-1-1-1-1-1-1	Edozighi	1278	Ibadan	5145	75
232	IR 88638-323-1-1-1-1-1-1	Edozighi	1420	Ibadan	5726	75
260	IR 88638-35-1-1-1-1-1-1	Edozighi	1431	Ibadan	5776	75
167	IR 88638-258-1-1-1-1-1-1	Edozighi	919	Ibadan	3713	75
225	IR 88638-314-1-1-1-1-1-1	Edozighi	1652	Ibadan	6711	75
45	IR 88638-140-1-1-1-1-1-1	Edozighi	1837	Ibadan	7470	75
125	IR 88638-216-1-1-1-1-1-1	Edozighi	1605	Ibadan	6537	75
208	IR 88638-296-1-1-1-1-1-1	Edozighi	1997	Ibadan	8140	75
22	IR 88638-117-1-1-1-1-1-1	Edozighi	2326	Ibadan	9588	76
56	IR 88638-151-1-1-1-1-1-1	Edozighi	1649	Ibadan	6815	76
266	IR 88638-4-1-1-1-1-1-1	Edozighi	1594	Ibadan	6600	76
235	IR 88638-326-1-1-1-1-1-1	Edozighi	1094	Ibadan	4539	76
322	IR 88638-95-1-1-1-1-1-1	Edozighi	1399	Ibadan	5811	76
230	IR 88638-321-1-1-1-1-1-1	Edozighi	1146	Ibadan	4762	76
326	IR 88638-99-1-1-1-1-1-1	Edozighi	1201	Ibadan	4998	76
129	IR 88638-220-1-1-1-1-1-1	Edozighi	1933	Ibadan	8087	76
60	IR 88638-155-1-1-1-1-1-1	Edozighi	1541	Ibadan	6457	76
271	IR 88638-46-1-1-1-1-1-1	Edozighi	1854	Ibadan	7778	76
98	IR 88638-192-1-1-1-1-1-1	Edozighi	1940	Ibadan	8161	76
211	IR 88638-300-1-1-1-1-1-1	Edozighi	1620	Ibadan	6862	76
306	IR 88638-80-1-1-1-1-1-1	Edozighi	1240	Ibadan	5253	76
315	IR 88638-89-1-1-1-1-1-1	Edozighi	1658	Ibadan	7041	76
187	IR 88638-276-1-1-1-1-1-1	Edozighi	1594	Ibadan	6795	77
299	IR 88638-73-1-1-1-1-1-1	Edozighi	1094	Ibadan	4674	77
91	IR 88638-185-1-1-1-1-1-1	Edozighi	1449	Ibadan	6187	77
156	IR 88638-248-1-1-1-1-1-1	Edozighi	1097	Ibadan	4687	77
168	IR 88638-259-1-1-1-1-1-1	Edozighi	1379	Ibadan	5906	77

117	IR 88638-210-1-1-1-1-1	Edozighi	1074	Ibadan	4608	77
308	IR 88638-81-1-1-1-1-1	Edozighi	1514	Ibadan	6518	77
4	IR 88638-10-1-1-1-1-1	Edozighi	1273	Ibadan	5504	77
185	IR 88638-274-1-1-1-1-1	Edozighi	1941	Ibadan	8420	77
291	IR 88638-66-1-1-1-1-1	Edozighi	1385	Ibadan	6042	77
49	IR 88638-144-1-1-1-1-1	Edozighi	1314	Ibadan	5760	77
275	IR 88638-50-1-1-1-1-1	Edozighi	1765	Ibadan	7736	77
224	IR 88638-313-1-1-1-1-1	Edozighi	1378	Ibadan	6039	77
106	IR 88638-20-1-1-1-1-1	Edozighi	1273	Ibadan	5591	77
77	IR 88638-172-1-1-1-1-1	Edozighi	1320	Ibadan	5800	77
276	IR 88638-5-1-1-1-1-1	Edozighi	1553	Ibadan	6826	77
217	IR 88638-305-1-1-1-1-1	Edozighi	1299	Ibadan	5728	77
71	IR 88638-166-1-1-1-1-1	Edozighi	1101	Ibadan	4858	77
32	IR 88638-127-1-1-1-1-1	Edozighi	1477	Ibadan	6547	77
206	IR 88638-294-1-1-1-1-1	Edozighi	1488	Ibadan	6593	77
101	IR 88638-195-1-1-1-1-1	Edozighi	1478	Ibadan	6572	78
293	IR 88638-68-1-1-1-1-1	Edozighi	1754	Ibadan	7816	78
1	BAO THAI	Edozighi	915	Ibadan	4083	78
131	IR 88638-221-1-1-1-1-1	Edozighi	1656	Ibadan	7391	78
52	IR 88638-147-1-1-1-1-1	Edozighi	1248	Ibadan	5590	78
307	IR 88638-8-1-1-1-1-1	Edozighi	1565	Ibadan	7060	78
35	IR 88638-130-1-1-1-1-1	Edozighi	1394	Ibadan	6299	78
171	IR 88638-261-1-1-1-1-1	Edozighi	1369	Ibadan	6192	78
239	IR 88638-33-1-1-1-1-1	Edozighi	1129	Ibadan	5110	78
288	IR 88638-63-1-1-1-1-1	Edozighi	1498	Ibadan	6782	78
102	IR 88638-196-1-1-1-1-1	Edozighi	1500	Ibadan	6803	78
100	IR 88638-194-1-1-1-1-1	Edozighi	2029	Ibadan	9246	78
159	IR 88638-25-1-1-1-1-1	Edozighi	1300	Ibadan	5931	78
223	IR 88638-311-1-1-1-1-1	Edozighi	1755	Ibadan	8028	78
258	IR 88638-348-1-1-1-1-1	Edozighi	1382	Ibadan	6325	78
15	IR 88638-1-1-1-1-1-1	Edozighi	2105	Ibadan	9652	78
277	IR 88638-51-1-1-1-1-1	Edozighi	1599	Ibadan	7354	78
151	IR 88638-241-1-1-1-1-1	Edozighi	1347	Ibadan	6200	78
34	IR 88638-129-1-1-1-1-1	Edozighi	1408	Ibadan	6508	78
289	IR 88638-64-1-1-1-1-1	Edozighi	2114	Ibadan	9818	78
247	IR 88638-338-1-1-1-1-1	Edozighi	1537	Ibadan	7137	78
274	IR 88638-49-1-1-1-1-1	Edozighi	1784	Ibadan	8297	79
143	IR 88638-233-1-1-1-1-1	Edozighi	1055	Ibadan	4924	79
46	IR 88638-14-1-1-1-1-1	Edozighi	1258	Ibadan	5893	79
181	IR 88638-27-1-1-1-1-1	Edozighi	1402	Ibadan	6578	79
316	IR 88638-90-1-1-1-1-1	Edozighi	1313	Ibadan	6171	79
158	IR 88638-250-1-1-1-1-1	Edozighi	1135	Ibadan	5347	79
80	IR 88638-175-1-1-1-1-1	Edozighi	1384	Ibadan	6528	79
201	IR 88638-290-1-1-1-1-1	Edozighi	1561	Ibadan	7393	79
64	IR 88638-160-1-1-1-1-1	Edozighi	1642	Ibadan	7783	79
66	IR 88638-161-1-1-1-1-1	Edozighi	1469	Ibadan	6972	79
62	IR 88638-158-1-1-1-1-1	Edozighi	2245	Ibadan	10663	79

138	IR 88638-229-1-1-1-1-1-1	Edozighi	1196	Ibadan	5685	79
9	IR 88638-105-1-1-1-1-1-1	Edozighi	1695	Ibadan	8064	79
263	IR 88638-38-1-1-1-1-1-1	Edozighi	1286	Ibadan	6121	79
261	IR 88638-36-1-1-1-1-1-1	Edozighi	1053	Ibadan	5018	79
28	IR 88638-122-1-1-1-1-1-1	Edozighi	1029	Ibadan	4918	79
273	IR 88638-48-1-1-1-1-1-1	Edozighi	1365	Ibadan	6533	79
210	IR 88638-299-1-1-1-1-1-1	Edozighi	1385	Ibadan	6638	79
137	IR 88638-228-1-1-1-1-1-1	Edozighi	1501	Ibadan	7207	79
69	IR 88638-164-1-1-1-1-1-1	Edozighi	1439	Ibadan	6926	79
161	IR 88638-252-1-1-1-1-1-1	Edozighi	1152	Ibadan	5566	79
83	IR 88638-178-1-1-1-1-1-1	Edozighi	1237	Ibadan	5996	79
162	IR 88638-253-1-1-1-1-1-1	Edozighi	1387	Ibadan	6765	80
190	IR 88638-279-1-1-1-1-1-1	Edozighi	1386	Ibadan	6764	80
205	IR 88638-293-1-1-1-1-1-1	Edozighi	1363	Ibadan	6656	80
268	IR 88638-43-1-1-1-1-1-1	Edozighi	1076	Ibadan	5271	80
13	IR 88638-109-1-1-1-1-1-1	Edozighi	1472	Ibadan	7231	80
313	IR 88638-86-1-1-1-1-1-1	Edozighi	1275	Ibadan	6293	80
153	IR 88638-245-1-1-1-1-1-1	Edozighi	1468	Ibadan	7255	80
147	IR 88638-238-1-1-1-1-1-1	Edozighi	2038	Ibadan	10153	80
44	IR 88638-138-1-1-1-1-1-1	Edozighi	1073	Ibadan	5352	80
116	IR 88638-209-1-1-1-1-1-1	Edozighi	1290	Ibadan	6448	80
54	IR 88638-150-1-1-1-1-1-1	Edozighi	1625	Ibadan	8129	80
216	IR 88638-304-1-1-1-1-1-1	Edozighi	1265	Ibadan	6348	80
120	IR 88638-211-1-1-1-1-1-1	Edozighi	1317	Ibadan	6625	80
59	IR 88638-154-1-1-1-1-1-1	Edozighi	1157	Ibadan	5824	80
183	IR 88638-272-1-1-1-1-1-1	Edozighi	1239	Ibadan	6259	80
24	IR 88638-119-1-1-1-1-1-1	Edozighi	1356	Ibadan	6888	80
309	IR 88638-82-1-1-1-1-1-1	Edozighi	1186	Ibadan	6027	80
231	IR 88638-322-1-1-1-1-1-1	Edozighi	839	Ibadan	4265	80
51	IR 88638-146-1-1-1-1-1-1	Edozighi	1113	Ibadan	5656	80
186	IR 88638-275-1-1-1-1-1-1	Edozighi	1783	Ibadan	9068	80
179	IR 88638-269-1-1-1-1-1-1	Edozighi	1192	Ibadan	6075	80
29	IR 88638-124-1-1-1-1-1-1	Edozighi	1902	Ibadan	9712	80
76	IR 88638-171-1-1-1-1-1-1	Edozighi	933	Ibadan	4783	80
55	IR 88638-15-1-1-1-1-1-1	Edozighi	1294	Ibadan	6653	81
177	IR 88638-267-1-1-1-1-1-1	Edozighi	1418	Ibadan	7295	81
108	IR 88638-202-1-1-1-1-1-1	Edozighi	1217	Ibadan	6273	81
303	IR 88638-77-1-1-1-1-1-1	Edozighi	1629	Ibadan	8402	81
33	IR 88638-128-1-1-1-1-1-1	Edozighi	1208	Ibadan	6310	81
81	IR 88638-176-1-1-1-1-1-1	Edozighi	1148	Ibadan	6007	81
194	IR 88638-282-1-1-1-1-1-1	Edozighi	850	Ibadan	4447	81
193	IR 88638-281-1-1-1-1-1-1	Edozighi	1212	Ibadan	6380	81
42	IR 88638-136-1-1-1-1-1-1	Edozighi	1856	Ibadan	9798	81
134	IR 88638-225-1-1-1-1-1-1	Edozighi	1430	Ibadan	7563	81
154	IR 88638-246-1-1-1-1-1-1	Edozighi	1150	Ibadan	6101	81
173	IR 88638-263-1-1-1-1-1-1	Edozighi	1271	Ibadan	6752	81
233	IR 88638-324-1-1-1-1-1-1	Edozighi	1448	Ibadan	7693	81

118	IR 88638-2-1-1-1-1-1	Edozighi	1598	Ibadan	8498	81
284	IR 88638-59-1-1-1-1-1	Edozighi	1296	Ibadan	6902	81
294	IR 88638-69-1-1-1-1-1	Edozighi	1122	Ibadan	6017	81
278	IR 88638-52-1-1-1-1-1	Edozighi	1013	Ibadan	5433	81
126	IR 88638-217-1-1-1-1-1	Edozighi	1138	Ibadan	6131	81
196	IR 88638-284-1-1-1-1-1	Edozighi	1178	Ibadan	6363	81
53	IR 88638-148-1-1-1-1-1	Edozighi	1523	Ibadan	8231	81
74	IR 88638-169-1-1-1-1-1	Edozighi	1104	Ibadan	5972	82
265	IR 88638-40-1-1-1-1-1	Edozighi	1364	Ibadan	7383	82
207	IR 88638-295-1-1-1-1-1	Edozighi	1192	Ibadan	6453	82
23	IR 88638-118-1-1-1-1-1	Edozighi	478	Ibadan	2609	82
197	IR 88638-285-1-1-1-1-1	Edozighi	901	Ibadan	4934	82
19	IR 88638-114-1-1-1-1-1	Edozighi	1237	Ibadan	6797	82
320	IR 88638-93-1-1-1-1-1	Edozighi	1090	Ibadan	5991	82
166	IR 88638-257-1-1-1-1-1	Edozighi	1668	Ibadan	9172	82
12	IR 88638-108-1-1-1-1-1	Edozighi	1862	Ibadan	10283	82
327	IR64	Edozighi	620	Ibadan	3439	82
157	IR 88638-249-1-1-1-1-1	Edozighi	1245	Ibadan	6903	82
99	IR 88638-193-1-1-1-1-1	Edozighi	1076	Ibadan	5994	82
61	IR 88638-156-1-1-1-1-1	Edozighi	902	Ibadan	5058	82
227	IR 88638-316-1-1-1-1-1	Edozighi	1068	Ibadan	5998	82
259	IR 88638-349-1-1-1-1-1	Edozighi	1202	Ibadan	6761	82
79	IR 88638-174-1-1-1-1-1	Edozighi	1476	Ibadan	8314	82
93	IR 88638-188-1-1-1-1-1	Edozighi	888	Ibadan	5008	82
17	IR 88638-112-1-1-1-1-1	Edozighi	1170	Ibadan	6605	82
280	IR 88638-55-1-1-1-1-1	Edozighi	1164	Ibadan	6585	82
214	IR 88638-302-1-1-1-1-1	Edozighi	1553	Ibadan	8813	82
141	IR 88638-231-1-1-1-1-1	Edozighi	1199	Ibadan	6814	82
21	IR 88638-116-1-1-1-1-1	Edozighi	1182	Ibadan	6745	82
78	IR 88638-173-1-1-1-1-1	Edozighi	1074	Ibadan	6134	82
90	IR 88638-184-1-1-1-1-1	Edozighi	1290	Ibadan	7373	83
88	IR 88638-182-1-1-1-1-1	Edozighi	1356	Ibadan	7792	83
18	IR 88638-113-1-1-1-1-1	Edozighi	1265	Ibadan	7270	83
204	IR 88638-292-1-1-1-1-1	Edozighi	1282	Ibadan	7375	83
86	IR 88638-18-1-1-1-1-1	Edozighi	1276	Ibadan	7367	83
123	IR 88638-214-1-1-1-1-1	Edozighi	1307	Ibadan	7544	83
241	IR 88638-332-1-1-1-1-1	Edozighi	1218	Ibadan	7057	83
11	IR 88638-107-1-1-1-1-1	Edozighi	1132	Ibadan	6565	83
175	IR 88638-265-1-1-1-1-1	Edozighi	854	Ibadan	4967	83
121	IR 88638-212-1-1-1-1-1	Edozighi	1143	Ibadan	6658	83
3	IR 88638-100-1-1-1-1-1	Edozighi	1443	Ibadan	8418	83
20	IR 88638-115-1-1-1-1-1	Edozighi	1110	Ibadan	6482	83
311	IR 88638-84-1-1-1-1-1	Edozighi	1212	Ibadan	7088	83
285	IR 88638-6-1-1-1-1-1	Edozighi	963	Ibadan	5669	83
85	IR 88638-180-1-1-1-1-1	Edozighi	1312	Ibadan	7733	83
245	IR 88638-336-1-1-1-1-1	Edozighi	1227	Ibadan	7361	83
152	IR 88638-244-1-1-1-1-1	Edozighi	1070	Ibadan	6436	83

295	IR 88638-70-1-1-1-1-1	Edozighi	841	Ibadan	5069	83
105	IR 88638-199-1-1-1-1-1	Edozighi	1154	Ibadan	6981	83
40	IR 88638-134-1-1-1-1-1	Edozighi	1041	Ibadan	6300	83
195	IR 88638-283-1-1-1-1-1	Edozighi	1184	Ibadan	7186	84
135	IR 88638-226-1-1-1-1-1	Edozighi	1686	Ibadan	10377	84
113	IR 88638-207-1-1-1-1-1	Edozighi	1088	Ibadan	6727	84
122	IR 88638-213-1-1-1-1-1	Edozighi	1183	Ibadan	7440	84
222	IR 88638-3-1-1-1-1-1	Edozighi	1340	Ibadan	8523	84
236	IR 88638-327-1-1-1-1-1	Edozighi	934	Ibadan	5956	84
290	IR 88638-65-1-1-1-1-1	Edozighi	629	Ibadan	4030	84
192	IR 88638-28-1-1-1-1-1	Edozighi	1236	Ibadan	7928	84
112	IR 88638-206-1-1-1-1-1	Edozighi	576	Ibadan	3712	84
130	IR 88638-22-1-1-1-1-1	Edozighi	1180	Ibadan	7742	85
200	IR 88638-288-1-1-1-1-1	Edozighi	1188	Ibadan	7842	85
133	IR 88638-224-1-1-1-1-1	Edozighi	1263	Ibadan	8353	85
296	IR 88638-7-1-1-1-1-1	Edozighi	807	Ibadan	5435	85
176	IR 88638-266-1-1-1-1-1	Edozighi	1048	Ibadan	7064	85
321	IR 88638-94-1-1-1-1-1	Edozighi	914	Ibadan	6379	86
323	IR 88638-96-1-1-1-1-1	Edozighi	976	Ibadan	6896	86
219	IR 88638-307-1-1-1-1-1	Edozighi	1345	Ibadan	9524	86
300	IR 88638-74-1-1-1-1-1	Edozighi	1272	Ibadan	9049	86
14	IR 88638-110-1-1-1-1-1	Edozighi	1188	Ibadan	8583	86
16	IR 88638-11-1-1-1-1-1	Edozighi	828	Ibadan	6080	86
292	IR 88638-67-1-1-1-1-1	Edozighi	748	Ibadan	5644	87
119	IR 88638-21-1-1-1-1-1	Edozighi	1273	Ibadan	10086	87
68	IR 88638-163-1-1-1-1-1	Edozighi	923	Ibadan	7359	87
272	IR 88638-47-1-1-1-1-1	Edozighi	1061	Ibadan	8470	87
65	IR 88638-16-1-1-1-1-1	Edozighi	1097	Ibadan	8848	88
189	IR 88638-278-1-1-1-1-1	Edozighi	613	Ibadan	4964	88
84	IR 88638-179-1-1-1-1-1	Edozighi	1071	Ibadan	9068	88
70	IR 88638-165-1-1-1-1-1	Edozighi	1136	Ibadan	9730	88
57	IR 88638-152-1-1-1-1-1	Edozighi	903	Ibadan	7773	88
172	IR 88638-262-1-1-1-1-1	Edozighi	986	Ibadan	8566	88
109	IR 88638-203-1-1-1-1-1	Edozighi	950	Ibadan	8259	88
72	IR 88638-167-1-1-1-1-1	Edozighi	1049	Ibadan	9168	89
103	IR 88638-197-1-1-1-1-1	Edozighi	783	Ibadan	6890	89
282	IR 88638-57-1-1-1-1-1	Edozighi	578	Ibadan	5116	89
202	IR 88638-29-1-1-1-1-1	Edozighi	1041	Ibadan	9312	89
209	IR 88638-298-1-1-1-1-1	Edozighi	724	Ibadan	6545	89
104	IR 88638-198-1-1-1-1-1	Edozighi	654	Ibadan	5943	89
240	IR 88638-331-1-1-1-1-1	Edozighi	868	Ibadan	7948	89
124	IR 88638-215-1-1-1-1-1	Edozighi	378	Ibadan	3650	90
174	IR 88638-264-1-1-1-1-1	Edozighi	863	Ibadan	8367	90
150	IR 88638-24-1-1-1-1-1	Edozighi	801	Ibadan	8164	90
283	IR 88638-58-1-1-1-1-1	Edozighi	791	Ibadan	8073	90
48	IR 88638-143-1-1-1-1-1	Edozighi	603	Ibadan	6480	91
279	IR 88638-53-1-1-1-1-1	Edozighi	533	Ibadan	9660	94
